# Targeting the Interaction of GABA_B_ Receptors With CHOP After an Ischemic Insult Restores Receptor Expression and Inhibits Progressive Neuronal Death

**DOI:** 10.3389/fphar.2022.870861

**Published:** 2022-03-29

**Authors:** Musadiq A. Bhat, Abolghasem Esmaeili, Elena Neumann, Karthik Balakrishnan, Dietmar Benke

**Affiliations:** ^1^ Institute of Pharmacology and Toxicology, University of Zurich, Zurich, Switzerland; ^2^ Department of Cell and Molecular Biology & Microbiology, Faculty of Biological Science and Technology, University of Isfahan, Isfahan, Iran; ^3^ Neuroscience Center Zurich, University of Zurich and ETH Zurich, Zurich, Switzerland; ^4^ Drug Discovery Network Zurich (DDNZ), Zurich, Switzerland

**Keywords:** GABA_B_ receptor, neuroprotection, interfering peptide, CHOP, cerebral ischemia, oxygen and glucose deprivation (OGD)

## Abstract

GABA_B_ receptors control neuronal excitability via slow and prolonged inhibition in the central nervous system. One important function of GABA_B_ receptors under physiological condition is to prevent neurons from shifting into an overexcitation state which can lead to excitotoxic death. However, under ischemic conditions, GABA_B_ receptors are downregulated, fostering over-excitation and excitotoxicity. One mechanism downregulating GABA_B_ receptors is mediated via the interaction with the endoplasmic reticulum (ER) stress-induced transcription factor CHOP. In this study, we investigated the hypothesis that preventing the interaction of CHOP with GABA_B_ receptors after an ischemic insult restores normal expression of GABA_B_ receptors and reduces neuronal death. For this, we designed an interfering peptide (R2-Pep) that restored the CHOP-induced downregulation of cell surface GABA_B_ receptors in cultured cortical neurons subjected to oxygen and glucose deprivation (OGD). Administration of R2-Pep after OGD restored normal cell surface expression of GABA_B_ receptors as well as GABA_B_ receptor-mediated inhibition. As a result, R2-Pep reduced enhanced neuronal activity and inhibited progressive neuronal death in OGD stressed cultures. Thus, targeting diseases relevant protein-protein interactions might be a promising strategy for developing highly specific novel therapeutics.

## 1 Introduction

Ischemic stroke is one of the leading causes of mortality and disability worldwide (Katan and Luft 2018). During cerebral ischemia reduced or blocked blood circulation deprives neurons from oxygen and glucose, which induces various destructive cellular reactions leading to neuronal death (White et al., 2000). The main cause of progressive neuronal death after an ischemic insult is neuronal overexcitation, resulting from aberrant activation of glutamate receptors and a massive accumulation of intracellular Ca^2+^ (excitotoxicity). Currently, there is no pharmacological treatment available for clinical use to inhibit progressive neuronal death after an ischemic insult.

Under physiological conditions, neuronal excitability is controlled by GABA_B_ receptors, preventing neurons to reach a state of overexcitation and thereby excitotoxicity (Chalifoux and Carter 2011). GABA_B_ receptors are heterodimeric G protein-coupled receptors consisting of GABA_B1_ and GABA_B2_ subunits. They are expressed in virtually all neurons at pre- and postsynaptic locations (Gassmann and Bettler 2012; Terunuma 2018). The binding of the neurotransmitter GABA to the receptor activates G_i/o_ proteins, which in turn modulate several effector systems inducing long-lasting neuronal inhibition. The most prominent effect of GABA_B_ receptors located at postsynaptic sites is the activation of G protein-coupled inwardly rectifying potassium (GIRK or K_ir_3) channels, resulting in the hyperpolarization of the neuronal membrane thereby reducing the likelihood of action potential generation (Gähwiler and Brown 1985; Luscher et al., 1997; Chalifoux and Carter 2011). At presynaptic sites the most obvious effect of GABA_B_ receptors is the inhibition of voltage-gated Ca^2+^ channels, thereby reducing neurotransmitter release (Mintz and Bean 1993; Santos et al., 1995; Chen and van den Pol 1998; Bussieres and El Manira 1999; Chalifoux and Carter 2011).

There is substantial *in vitro* and *in vivo* evidence for downregulation of GABA_B_ receptors under conditions leading to neuronal overexcitation and neuronal death (Vollenweider et al., 2006; Guetg et al., 2010; Maier et al., 2010; Terunuma et al., 2010; Kim et al., 2011; Kantamneni et al., 2014; Maier et al., 2014; Huang et al., 2017; Hleihil et al., 2021). This unfortunate situation removes GABA_B_ receptors as “emergency brake” to combat neuronal overexcitation from the system and further fosters excitotoxic neuronal death. One mechanism that downregulates functional GABA_B_ receptors, i. e. receptors expressed at the cell surface, involves the endoplasmic reticulum (ER) stress-induced transcription factor C/EBP homologous protein (CHOP) (Maier et al., 2014). CHOP, also known as C/EBPζ, growth arrest and DNA damage-inducible gene 153 (GADD153) or DNA-damage-inducible transcript 3 (DDIT3), belongs to the CCAAT/enhancer-binding proteins (C/EBP) family and is only marginally expressed under physiological conditions (Oyadomari and Mori 2004; Yang et al., 2017; Hu et al., 2018). However, CHOP is strongly upregulated upon ER stress associated with ischemia and induces apoptotic cell death (Zinszner et al., 1998; Oyadomari and Mori 2004; Morimoto et al., 2007; Oida et al., 2008).

In addition to its pro-apoptotic activity, upregulated CHOP interacts via its C-terminal leucine zipper with the leucine zipper within the C-terminal domain of GABA_B2_ (Sauter et al., 2005). This interaction blocks the assembly of the GABA_B_ receptor subunits GABA_B1_ and GABA_B2_ in the ER and thereby prevent the exit of assembled receptors from ER (Maier et al., 2014). Because the supply of newly formed GABA_B_ receptors is inhibited, ongoing constitutive internalization and lysosomal degradation of cell surface receptors downregulates functional receptors and thereby GABA_B_ receptor-mediated inhibition (Maier et al., 2014).

Enhancing GABA_B_ receptor activity using the agonist baclofen showed beneficial effects in *in vivo* and *in vitro* models of cerebral ischemia (Lal et al., 1995; Jackson-Friedman et al., 1997; Kulinskii and Mikhel’son 2000; Babcock et al., 2002; Dave et al., 2005; Zhang et al., 2007; Xu et al., 2008; Cimarosti et al., 2009; Liu et al., 2015; Hleihil et al., 2021). These observations provide strong support that GABA_B_ receptors may constitute valid targets for a neuroprotective strategy. As GABA_B_ receptors are downregulated under ischemic conditions, activation of the receptors by baclofen might have only a limited neuroprotective effect. We, therefore, tested the hypothesis that restoring normal cell surface GABA_B_ receptor expression in neurons subjected to ischemic stress by preventing its interaction with CHOP would reduce ischemia-induced neuronal overexcitation and diminish neuronal death. To inhibit the interaction of GABA_B_ receptors with CHOP, we designed a short interfering peptide (R2-Pep) that comprises a specific sequence corresponding partially to the coiled-coil domain of GABA_B2_, which is part of the binding site for CHOP (Sauter et al., 2005). R2-Pep indeed restored cell surface expression of GABA_B_ receptors in cultured cortical neurons subjected to OGD (mimicking cerebral ischemia) and inhibited progressive neuronal death.

## 2 Materials and Methods

### 2.1 Neuron-Glia Co-cultures

The use of rat embryos for generating primary neuronal cultures was approved by the Zurich cantonal veterinary office, Zurich, Switzerland (licence ZH011/19). All cell culture media used were from Gibco. The cerebral cortex of 18 days old rat embryos were carefully dissected and washed with 5 ml sterile-filtered PBGA buffer (PBS containing 10 mM glucose, 1 mg/ml bovine serum albumin and antibiotic-antimycotic 1:100 (10,000 units/ml penicillin; 10,000 μg/ml streptomycin; 25 μg/ml amphotericin B)). The cortices were cut into small pieces with a sterile scalpel and digested in 5 ml sterile filtered papain solution for 15 min at 37°C. The supernatant was removed and the tissue was washed twice with complete DMEM/FCS medium (Dulbecco’s Modified Eagle’s Medium containing 10% Fetal Calf Serum and penicillin/streptomycin, 1:100). Then, fresh DMEM/FCS was added, and the tissue was gently triturated and subsequently filtered through a 40 μm cell-strainer. Finally, the neurons were plated at a concentration of 60,000–80,000 cells per well onto the poly-l-lysine (50 μg/ml in PBS) coated coverslips in a 12-well culture dish and incubated overnight at 37°C and 5% CO_2_. After 24 h of incubation, the DMEM medium was exchanged with freshly prepared NU-medium (Minimum Essential Medium (MEM) with 15% NU serum, 2% B27 supplement, 15 mM HEPES, 0.45% glucose, 1 mM sodium pyruvate, 2 mM GlutaMAX). The cultures were kept for 12–16 days *in vitro*.

### 2.2 Oxygen Glucose Deprivation Stress

The OGD medium (DMEM lacking glucose, glutamine, sodium pyruvate, HEPES and phenol red) was deprived from oxygen by equilibrating it with nitrogen for 15 min in a water bath at 37°C. 1 ml of equilibrated OGD medium was added into each well of a 12-well culture plate and the coverslips containing the cultured neurons were transferred to the OGD medium. The culture plate was then incubated for 1 hour in a hypoxic incubator at 1% O_2_, 5% CO_2_ and 37°C. The coverslips were then transferred back to the culture plate containing the original conditioned culture medium and incubated at 37°C and 5% CO_2_. Unless otherwise stated, neurons were analyzed after a recovery period of 16 h.

### 2.3 Interfering Peptide

The interfering peptide (R2-Pep) comprises a sequence derived partially from the coiled-coil domain of GABA_B2_ (LQDTPEKTTYIK). A peptide (Ctrl-Pep) containing the same amino acids but in a random sequence (PETKTDLTQYKI) was used as a control (Ctrl-Pep). To render the peptides cell-permeable, they were tagged at their N-terminus with the protein transduction domain of trans-activator of transcription (TAT) followed by an arginine-rich region (GRKKRRQRRRPQRRRRRRRR). Both peptides were custom synthesized at PEPMIC Co., Ltd., Suzhou, China and labelled at the N-terminus with FITC.

For all experiments, except for the survival assay, the cultures were treated with R2-Pep (10 μg/ml) or with Ctrl-Pep (10 μg/ml) in their original culture medium immediately after the OGD incubation and then incubated for 16 h at 37°C and 5% CO_2_.

### 2.4 Immunofluorescence Staining

For cell surface staining of GABA_B_ receptors, an antibody directed against the N-terminus of GABA_B2_ (GABA_B2_N, custom-made by GeneScript) was used (Benke et al., 2002)). Coverslips containing the cultured neuron/glia cells were washed 3 times with cold buffer A (25 mM HEPES pH 7.4, 119 mM NaCl, 2.5 mM KCl, 2 mM CaCl_2_, 1 mM MgCl_2_ and 30 mM glucose). Then the GABA_B2_N antibody (1:250 dilution in buffer A containing 10% normal donkey serum (NDS)) was added and incubated on ice for 90 min. The coverslips were then washed 3 times for 5 min with buffer A, followed by incubation with Cy3-labelled donkey anti-rabbit secondary antibody (1:400 in PBS/10% NDS, Jackson ImmunoResearch) for 60 min on ice. Afterwards, the coverslips were washed again 3 times for 5 min with buffer A.

For subsequent staining of intracellular CHOP, the cells were fixed with 4% PFA for 30 min at room temperature. After fixation, the cells were washed with PBS and permeabilized by incubation for 12 min in 0.2% Triton X-100/PBS. Then, CHOP/GADD 153 antibody (1:500, clone B-3, Cat No. sc-7351, Santa Cruz) was added and incubated overnight at 4°C. After incubation, the coverslips were washed 3 times for 5 min with PBS. Then, Cy3-or Cy5-labeled donkey anti-mouse antibody (dilution 1:400 in PBS/10% NDS, Jackson ImmunoResearch) was added and incubated for 1 h at room temperature. Finally, the coverslips were washed again 3 times for 5 min with PBS and mounted in DAKO fluorescence mounting medium onto glass slides for microscopy.

For monitoring K_ir_3.2 channel expression, the coverslips were washed with PBS, fixed for 30 min in 4% PFA, followed by permeabilization with 0.2% Triton X-100 in PBS for 12 min and incubation with anti-K_ir_3.2 antibody (1:250, Cat No. APC-006, Alomone Labs). Antibody incubation and further treatment were done as described for staining of CHOP.

### 2.5 *In situ* Proximity Ligation Assay

*In situ* PLA was used to investigate the interaction between GABA_B1_ and GABA_B2_ subunits as well as CHOP and R2-Pep. The *in situ* PLA was performed using the Duolink II kit (Sigma Aldrich) according to the instructions of the manufacturer. Briefly, the neurons were washed with PBS and then fixed with 4% PFA for 30 min at room temperature. Then the coverslips were rinsed in PBS for 5 min and permeabilized for 12 min with 0.2% Triton X-100/PBS. After rinsing the coverslips in PBS, they were incubated with the primary antibody solution consisting of a rabbit antibody raised against the C-terminal domain of GABA_B2_ (1:250, Cat. No. ab75838, Abcam) and a mouse antibody directed against GABA_B1_ (1:50, Cat No. ab55051, Abcam) or in case of the CHOP/R2-Pep interaction with mouse CHOP antibody (1:40, clone B-3, Cat. No. sc-7351, Santa Cruz) and goat FITC antibody (1:400, Cat. No. NB600-1273, Novus Biologicals; R2-Pep was labeled with FITC at the N-terminus) in a humidity chamber overnight at 4°C. Subsequently, the cultures were washed four times for 5 min with PBS and incubated for 20–30 min at room temperature with the PLA probes (prepared by diluting anti-Mouse MINUS and anti-Rabbit PLUS or anti-Goat PLUS (Duolink II) 1:5 in 5% BSA/PBS). Afterwards, 40 μL of the PLA probe solution were pipetted on top of each coverslip and incubated in a humidity chamber for 1 h at 37°C. The coverslips were then washed two times for 5 min in PBS and incubated for 1 h at 37°C with ligation solution. Subsequently, the cells were washed two times in Duolink II Wash Buffer A and incubated with the amplification solution at 37°C for 100 min. Finally, the coverslips were washed two times for 10 min with Duolink II Wash Buffer B in the dark and mounted onto microscope slides with DAKO fluorescent mounting medium.

### 2.6 Quantification of Neuronal Loss

To quantify neuronal death, co-cultures of neurons and glia cells subjected to OGD and subsequently treated with R2-Pep or Ctrl-Pep were stained with an antibody directed against the neuron-specific marker protein NeuN (1:400, Cat. No. ABN78, Millipore), followed by staining with Alexa Fluor Plus 488 secondary antibodies (1:2000, Jackson ImmunoResearch). The total number of cells was determined by counting the cell nuclei stained with DAPI included in the fluorescent mounting medium. After microscopy, neuronal loss was quantified by counting the number of neurons and number of DAPI positive nuclei using the ImageJ plugin “Cell Counter” and the ratio of neurons to total cells (DAPI positive nuclei) per image was calculated.

### 2.7 Microscopy and Image Analysis

Images were taken with a Zeiss laser scanning confocal microscope (CLSM700 or CLSM710) in sequential mode using the Zeiss 40x (1.3 NA) and 100x (1.45 NA) plan-fluar objective, with a resolution of 1024 × 1024 pixels. The laser intensity and the detector gain were adjusted to values that avoid signal saturation and all images of one experiment was imaged with the same settings in one continuous session. The images were quantitatively analysed using the software ImageJ.

For quantification of cell surface staining, the outer and the inner perimeter of the cell surface were exactly outlined. Then the fluorescence intensity value obtained from the inner border was subtracted from the one of the outer border so that only the fluorescence present at the cell surface was determined and used for statistical evaluation.

For quantification of the total cell staining, only the outer border of the cell was marked and the mean fluorescence intensity was measured.

For quantification of *in situ* PLA signals, the soma of neurons was surrounded and the fluorescent dots inside these borders were counted using the ImageJ option “Find maxima.” A fixed noise tolerance value was used for the analysis of all images of the same experiment. The PLA signals were normalized to the area analyzed.

### 2.8 Electrophysiological Experiments

Cortical neurons were recorded in the whole-cell voltage-clamp configuration at room temperature using a HEKA EPC10 amplifier and the Patchmaster software. Spontaneous postsynaptic currents (sPSCs) were recorded at a holding potential of -60 mV. Patch electrodes were filled with 120 mM CsCl or KCl, 10 mM EGTA, 10 mM HEPES pH 7.4, 4 mM MgCl_2_, 0.5 mM GTP and 2 mM ATP. Spontaneous PSCs recordings were performed using intracellular CsCl, whereas the potassium currents were recorded using an intracellular solution containing KCl. The external solution contained 140 mM NaCl, 10 mM KCl, 2 mM CaCl_2_, 1 mM MgCl_2_, 10 mM HEPES pH 7.4 and 10 mM glucose.

Potassium currents were recorded in the presence of tetrodotoxin (0.5 μM), 7-nitro-2,3-dioxo-1,4-dihydroquinoxaline-6-carbonitrile (CNQX, 2 μM), and bicuculline (4 μM). To enhance the amplitude of the baclofen-evoked currents, the potassium concentration of the extracellular solution was increased to 30 mM, and the sodium concentration was reduced to 120 mM (to keep osmolarity constant). GABA_B_ receptor-evoked potassium currents were elicited using a 10 s pulse of 50 μM baclofen at −90 mV.

All synaptic events displaying amplitudes above the background noise (5–12 pA) were identified and analyzed off-line using the MiniAnalysis 6.0.7 software (Synaptosoft). Mean amplitudes and frequency values were obtained from 1 min recording epochs on each experimental condition and normalized to the control condition of the individual neuron.

### 2.9 Statistics

The statistical evaluation of data was performed using the software GraphPad Prism (version 8.4.3.). All results were given as mean value ±standard error of the mean (SEM). The data were analyzed by one- or two-way ANOVA or Kruskal–Wallis test followed by appropriate post hoc tests as indicated in the figure legends. Data sets were tested for normal or lognormal distributions by the D’Agostino-Pearson test and QQ-plots. In case of significant deviation from homoscedasticity Welch and Brown Forsythe variations of ANOVA was used. A *p*-value of <0.05 was considered as statistically significant.

## 3 Results

### 3.1 Oxygen Glucose Deprivation Stress Leads to Immediate Upregulation of CHOP Expression and Downregulation of GABA_B_ Receptors

**FIGURE 1:**
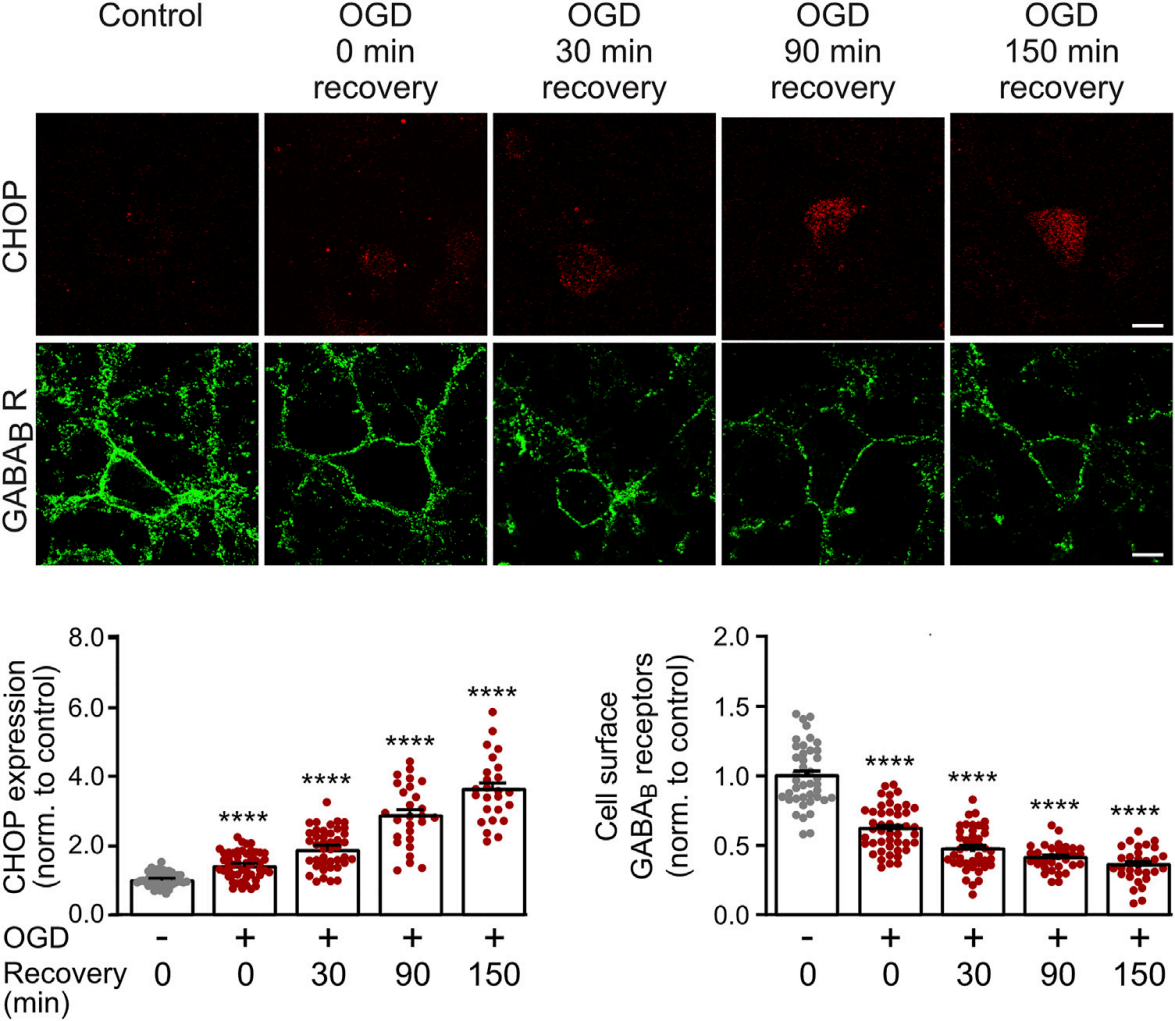
Time course of OGD-induced downregulation of GABA_B_ receptor cell surface expression and CHOP upregulation. Neurons were exposed to OGD for 1 h, returned to the normal culture medium and tested for GABA_B2_ cell surface as well as CHOP expression at different time intervals (0, 30, 90 and 150 min) thereafter. For the 0 min recovery time, the cultures were removed from the OGD medium, washed once in PBS for 2 min and then fixated. Top: representative images, the scale bar corresponds to 10 μm. Bottom: quantification of fluorescence intensities. Neurons not subjected to OGD served as a control. The data represent the mean ± S.E.M. of 31–46 neurons for each experimental condition derived from three independent experiments. ****, *p* ˂ 0.0001; Brown-Forsythe/Welch one-way ANOVA followed by Dunnett’s T3 multiple comparison test.

We previously showed that CHOP upregulation is maintained in cultured neurons for at least 24 h after being exposed to OGD stress (Maier et al., 2014). However, we did not test for the earliest time point after OGD at which a significant up-regulation of CHOP occurs that could affect GABA_B_ receptor cell surface expression. This information is important for selecting the optimal time point for the electrophysiological experiments, which should as early as possible after the OGD stress for technical reasons. We, therefore, subjected cultured neurons to OGD stress and monitored CHOP expression as well as GABA_B_ receptor cell surface levels either directly after OGD stress or after a recovery period of 30–150 min. Significant up-regulation of CHOP was visible immediately OGD stress and progressively increased thereafter (Figure 1). Conversely, cell surface expression of GABA_B_ receptors concomitantly decreased (Figure 1). These results show a correlated up- and down-regulation of CHOP and GABA_B_ receptors, respectively, directly after 1 h of OGD-induced stress interval.

### 3.2 Identification of a Peptide Interfering With the Stress-Induced Interaction of CHOP and GABA_B_ Receptors

**FIGURE 2:**
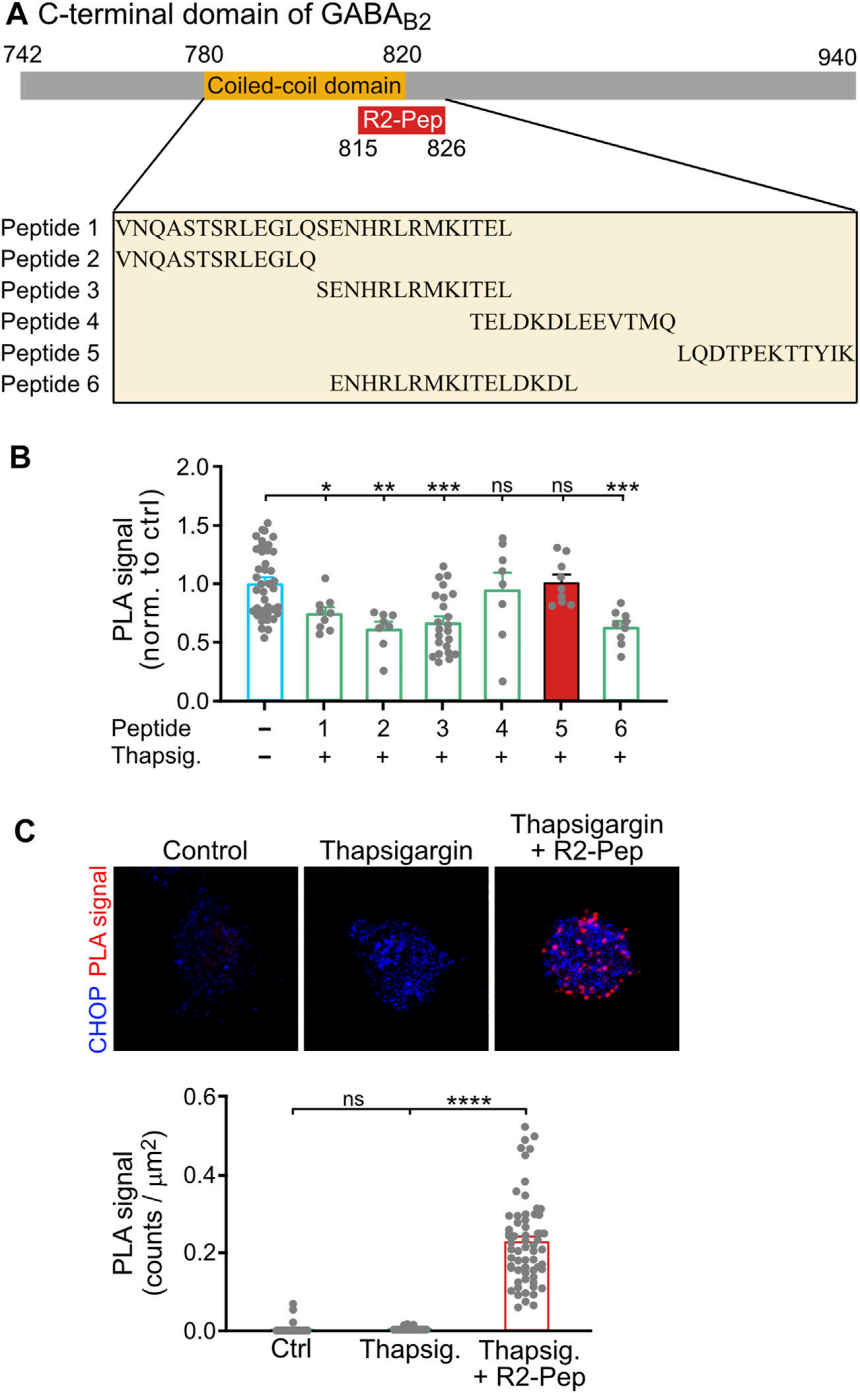
Screening for a peptide (R2-Pep) interfering with the interaction of CHOP with GABA_B_ receptors. **(A)** Scheme of the C-terminal domain of GABA_B2_ with the coiled-coil domain (orange box), which contributes to the CHOP-GABA_B_ receptor interaction site and location of the interfering peptide R2-Pep sequence (red box). The box below depicts the GABA_B2_ peptide sequences used for screening. **(B)** Peptide 5 reliably prevented stress-induced decrease in GABA_B1_/GABA_B2_ heteromers. Cultures were stressed for 2 h with thapsigargin (1 μM) and immediately thereafter treated for 30 min with the peptides indicated in **(A)**. Neurons were then tested for the interaction between GABA_B1_ and GABA_B2_ by *in situ* PLA. The *in situ* PLA signals of the untreated neurons served as control (-, no peptide). The data represent the mean ± S.E.M. of 8–40 neurons derived from two independent experiments (control, *n* = 4). ns, *p* > 0.05; *, *p* < 0.05; **, *p* < 0.005; ***; *p* < 0.0005. Brown-Forsythe/Welch one-way ANOVA followed by Games-Howell’s multiple comparison test. **(C)** R2-Pep (peptide 5 in **B**) interacted with CHOP. Cultures were stressed for 2 h with thapsigargin (1 μM) and immediately thereafter treated with R2-Pep for 30 min. Neurons were then tested for the interaction of CHOP with R2-Pep by *in situ* PLA using antibodies directed against CHOP and FITC (R2-Pep was labeled with FITC at the N-terminus). The data represent the mean ± S.E.M. of 47–62 neurons derived from two independent experiments. ns, *p* > 0.05; ****; *p* < 0.0001. Kruskal–Wallis test followed by Dunn’s multiple comparison test.

CHOP interacts with GABA_B_ receptors via the coiled-coil motif of GABA_B2_ located in the intracellularly located C-terminal domain (Sauter et al., 2005) and prevents heteromerization of GABA_B1_ with GABA_B2_ in the ER (Maier et al., 2014). For the identification of a small peptide interfering with this interaction, we designed a set of synthetic peptides covering the GABA_B2_ coiled-coil domain and tested them for their ability to restore ER stress-induced (1 μM thapsigargin for 2 h) impairment of heteromerization of GABA_B1_ and GABA_B2_ in cultured neurons using *in situ* PLA. The screening yielded one peptide comprising the GABA_B2_ sequence 815–826 (LQDTPEKTTYIK) that reliably restored the expression of GABA_B1_/GABA_B2_ heteromers (Figures 2A,B). This peptide was rendered cell-permeable by tagging it at the N-terminus with the HIV trans-activator of transcription (TAT) sequence followed by an arginine-rich region (this peptide was named R2-Pep). In addition, the peptide was labelled at the N-terminus with FITC for identification. R2-Pep interacted with upregulated CHOP (cultures treated with 1 μM thapsigargin for 2 h) as indicated by numerous *in situ* PLA signals (Figure 2C).

### 3.3 R2-Pep Rescued Expression of Cell Surface GABA_B_ Receptors After Oxygen Glucose Deprivation Stress and Reduced CHOP Expression

**FIGURE 3:**
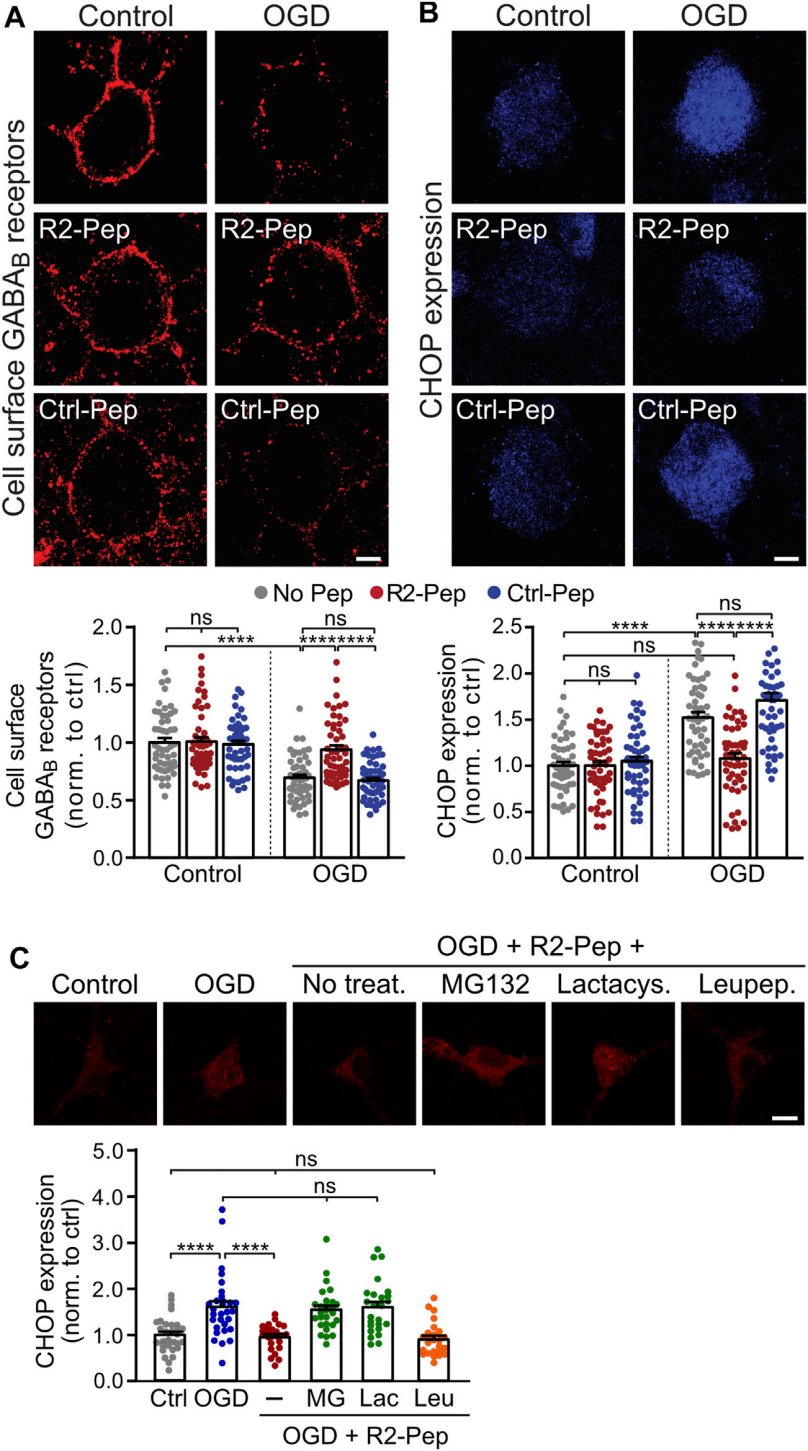
CHOP-mediated down-regulation of cell surface GABA_B_ receptors is rescued by R2-Pep. **(A)** and **(B)**, Cultures were stressed for 1 h with OGD and immediately thereafter treated with R2-Pep, the inactive Ctrl-Pep or remained untreated. The neurons were immunostained for cell surface GABA_B2_ **(A)** and CHOP **(B)** 16 h after OGD stress. Top: representative images (scale bar: 5 μm). Bottom: quantification of fluorescence intensities. The fluorescence intensity of the untreated neurons served as controls. The data represent the mean ± S.E.M. of 49–53 neurons for each experimental condition derived from three independent experiments. ns, *p* > 0.05; ****; *p* < 0.0001; two-way ANOVA with Tukey’s multiple comparisons test. **(C)** R2-Pep induces proteasomal degradation of CHOP upregulated by OGD. Cultures were stressed for 1 h with OGD and immediately thereafter remained untreated or were treated with R2-Pep in the presence of proteasome inhibitors (50 μM lactacystin or 50 μM MG132) or the lysosome inhibitor leupeptin (50 μM). The neurons were immunostained for CHOP expression 16 h after OGD stress. Top: representative images (scale bar: 10 μm). Bottom: quantification of fluorescence intensities. The fluorescence intensity of untreated neurons served as control. The data represent the mean ± S.E.M. of 25–30 neurons for each experimental condition derived from two independent experiments. ****, *p* < 0.0001; ns, *p* > 0.05; one-way ANOVA with Tukey’s multiple comparisons test.

Next, we tested for the ability of R2-Pep to normalize OGD-induced downregulation of GABA_B_ receptors. Treatment of neurons with R2-Pep immediately after OGD restored cell surface expression of GABA_B_ receptors to normal physiological levels (Figure 3A, tested after a recovery period of 16 h). Interestingly, treatment with R2-Pep also reversed the upregulated CHOP expression to normal levels (Figure 3B). As CHOP is constitutively polyubiquitinated and degraded by proteasomes (Hattori et al., 2003), the most likely explanation was the proteasomal degradation of the CHOP/R2-Pep complex. Indeed, inhibition of proteasomal degradation by lactacystin or MG132 completely abolished the downregulation of CHOP induced by R2-Pep, whereas inhibition of lysosomal degradation had no effect (Figure 3C).

Treatment of R2-Pep to neurons not subjected to OGD did neither affect basal CHOP expression nor cell surface GABA_B_ receptor expression. As expected, an inactive control peptide (Ctrl-Pep, same amino acids as R2-Pep but in a random order) did not affect GABA_B_ receptor and CHOP expression under control or OGD conditions (Figures 3A,B).

### 3.4 R2-Pep Restores GABA_B_ Receptor Heteromers in Oxygen Glucose Deprivation Stressed Neurons

**FIGURE 4:**
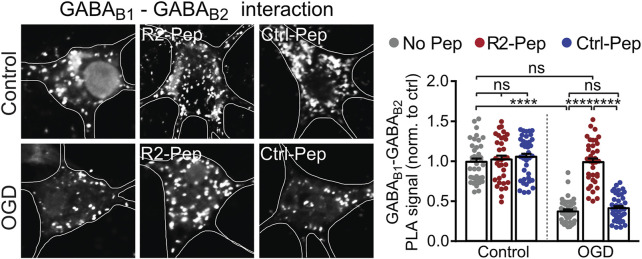
CHOP-mediated decrease of GABA_B_ receptor heterodimerization is rescued by R2-Pep. Cultures were stressed for 1 h with OGD and immediately thereafter treated with R2-Pep, the inactive Ctrl-Pep or remained untreated. After 3 h, the interaction between GABA_B1_ and GABA_B2_ was tested by *in situ* PLA. Left: representative images (scale bar: 5 μm). Right: quantification of the GABA_B1_-GABA_B2_ interaction in control or OGD treated neurons in the absence or presence of R2-Pep or Rand-Pep. The interaction level observed in untreated neurons served as control. The data represent the mean ± S.E.M. of 37–41 neurons for each experimental condition derived from three independent experiments. ****, *p* < 0.0001; ns: *p* > 0.05; two-way ANOVA with Tukey’s multiple comparisons test.

Up-regulated CHOP interacts with GABA_B_ receptors in the ER, prevents their heterodimerization and thereby inhibits their forward trafficking to the cell surface (Maier et al., 2014). To test whether R2-Pep normalizes the CHOP-induced impaired heterodimerization of GABA_B_ receptors, cultured neurons were subjected to OGD and analyzed for GABA_B1_-GABA_B2_ interaction by *in situ* PLA in the presence or absence of the interfering peptide R2-Pep or the inactive Ctrl-Pep (Figure 4). OGD stress significantly decreased GABA_B_ receptor heteromers in neurons, which was normalized to physiological levels (control) upon administration of R2-Pep. In contrast, treatment of neurons with R2-Pep not subjected to OGD did not affect the level of GABA_B_ receptors heteromers. In addition, the inactive control peptide did not affect the levels of GABA_B_ receptor heteromers under control or OGD conditions, indicating the specificity of R2-Pep (Figure 4).

### 3.5 R2-Pep Normalizes Oxygen Glucose Deprivation-Mediated Downregulation of K_ir_3.2 Channels

**FIGURE 5:**
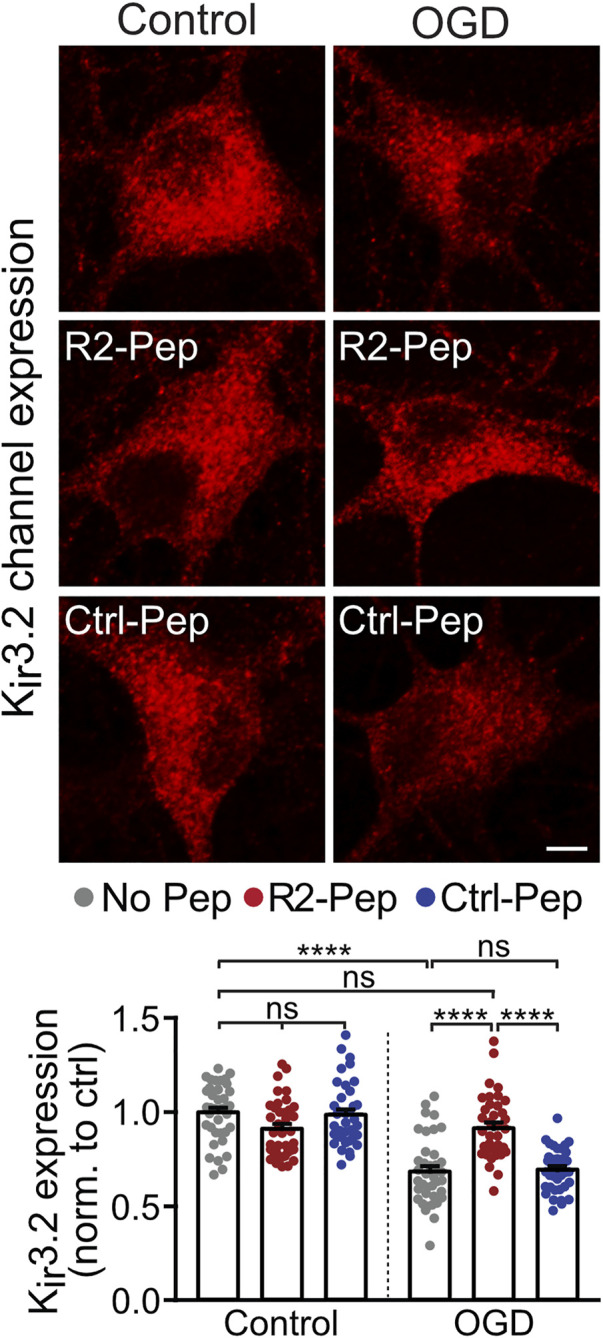
R2-Pep normalize OGD-mediated dysregulation of K_ir_3.2 channels. Cultures were stressed for 1 h with OGD and immediately treated with R2-Pep, Ctrl-Pep or remained untreated. After 16 h neurons were immunostained for K_ir_3.2 channel expression. Top: representative images (scale bar: 5 μm). Bottom: quantification of fluorescence intensities. The fluorescence intensity of the untreated neurons served as control. The data represent the mean ± S.E.M. of 37–38 neurons from two independent preparations. ns: *p* > 0.05; ****, *p* < 0.0001; two-way ANOVA with Tukey’s multiple comparisons test.

Postsynaptically, GABA_B_ receptor-mediated inhibition is mainly conveyed via activation of inwardly rectifying K^+^ channels (K_ir_3.2), leading to inhibitory postsynaptic potentials (Luscher et al., 1997). As GABA_B_ receptors form signalling complexes with their effector proteins (Schwenk et al., 2016), we expected a correlated regulation of the receptors with K_ir_3.2 channels. Therefore, we analyzed the effect of OGD and R2-Pep on the expression of K_ir_3.2 channels. In neurons stressed with OGD, K_ir_3.2 channels were downregulated (Figure 5), comparable to GABA_B_ receptors. Treatment with R2-Pep immediately after OGD restored normal expression levels of K_ir_3.2, being in line with a co-regulation of GABA_B_ receptors and K_ir_3.2 in signalling complexes. The inactive Ctrl-Pep did not affect expression of the effectors, documenting the specificity of R2-Pep (Figure 5). In addition, R2-Pep did not affect expression of the effectors under control conditions, i. e. unstressed neurons (Figure 5).

### 3.6 R2-Pep Restores GABA_B_ Receptor-Mediated Inhibition in Oxygen Glucose Deprivation Stressed Neurons and Thereby Reduces Enhanced Neuronal Activity

**FIGURE 6:**
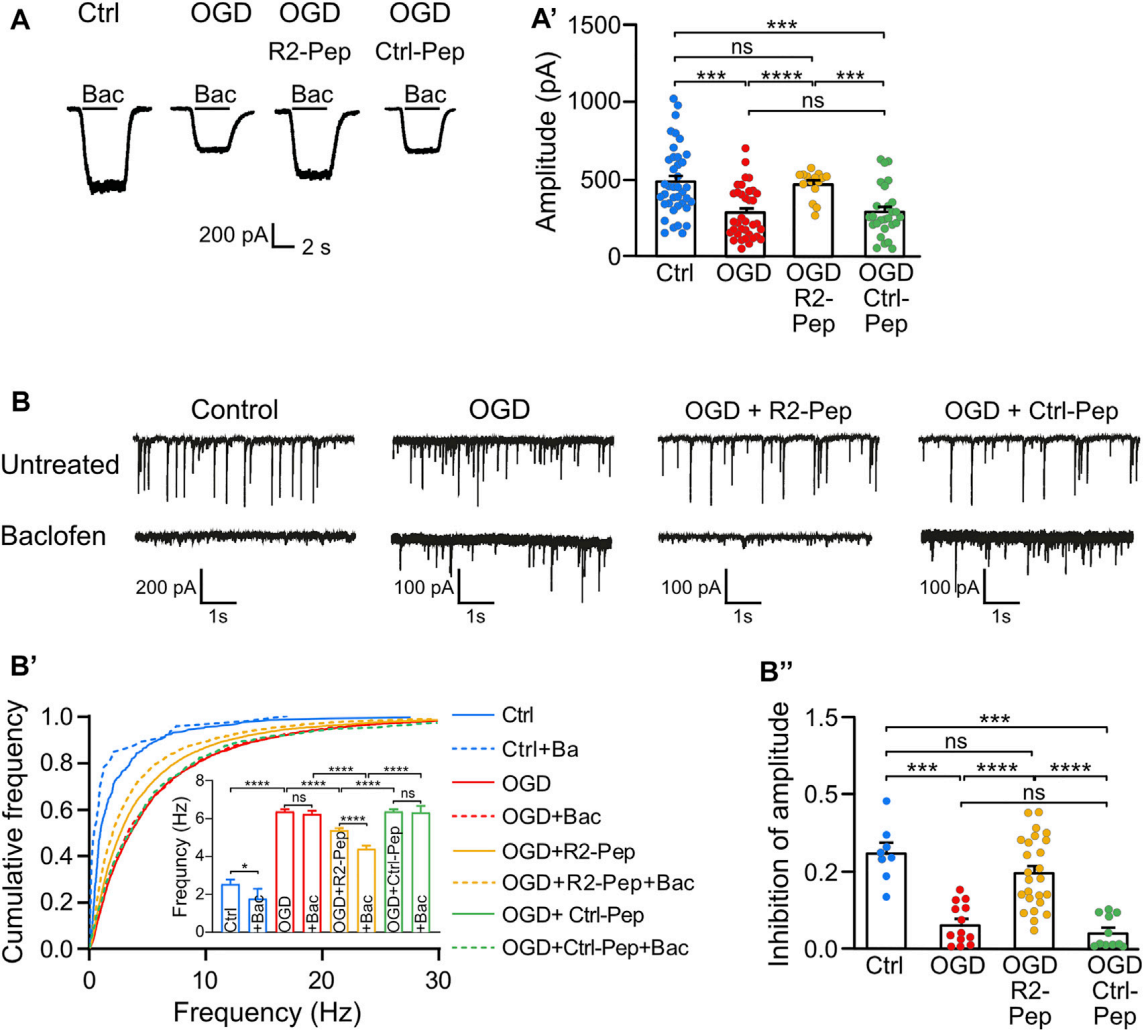
Treatment of OGD-stressed neurons with R2-Pep restores GABA_B_ receptor-mediated inhibition. **(A)** R2-Pep restored OGD-induced downregulated GABA_B_ receptor-mediated K^+^ currents. Representative traces of baclofen-induced K^+^ currents were recorded in untreated neurons or OGP-treated neurons in the presence or absence of R2-Pep or the inactive Ctrl-Pep. **(A′**), Quantitative evaluation of K^+^ current amplitudes. The data represent the mean ± S.E.M. of Ctrl: *n* = 39, OGD: *n* = 36, OGD + R2-Pep: *n* = 15, OGD + Ctrl-Pep: *n* = 27. Ns, *p* > 0.05; ***, *p* < 0.001 ****, *p* < 0.0001, Brown-Forsythe/Welch one-way ANOVA followed by Dunnett’s T3 multiple comparison test. **(B)** Treatment of OGD-stressed neurons with R2-Pep restores baclofen-induced inhibition of the amplitude and frequency of spontaneous postsynaptic currents (sPSCs). Representative current traces depicting sPSCs recorded from untreated neurons and OGD-stressed neurons in the absence or presence of R2-Pep or the inactive Ctrl-Pep. **(B′)**, Cumulative frequency histogram of inter-event-intervals of sPSCs recorded from cells without (solid lines) or with baclofen (dashed lines). Ns, *p* > 0.05; *, *p* < 0.05; ****, *p* < 0.0001; Kruskal–Wallis test followed by Dunn’s multiple comparison test (Ctrl: *n* = 8, OGD: *n* = 13, OGD + R2-Pep: *n* = 25, OGD + Ctrl: *n* = 11). **(B”)**, Inhibition of sPSC amplitudes by baclofen. Mean amplitudes values were normalized to the control condition of the individual neuron. Ns, *p* > 0.05; ***, *p* < 0.001; ****, *p* < 0.0001; Brown-Forsythe/Welch one-way ANOVA followed by Games-Howell’s multiple comparison test (Ctrl: *n* = 8, OGD: *n* = 13, OGD + R2-Pep: *n* = 25, OGD + Ctrl-Pep: *n* = 11).

Next, we assessed the functional consequences of restored GABA_B_ receptor levels after exposing cultured neurons to OGD in the presence or absence of R2-Pep by measuring baclofen-induced K^+^ currents using whole-cell patch-clamp recordings. As expected, we observed a strong reduction of baclofen-induced K^+^ currents amplitudes (i. e. GABA_B_ receptor-mediated currents) in OGD-stressed neurons, which were nearly completely restored to control amplitude values after treatment with R2-Pep (Figure 6A). In contrast, the control peptide (Ctrl-Pep) showed no effect. This finding supports the hypothesis that R2-Pep restores functional GABA_B_ receptors available for neuronal inhibition.

Next, we analyzed whether restored GABA_B_ receptor-mediated inhibition can reduce OGD-induced neuronal overexcitation. OGD treatment strongly increased the frequency of spontaneous postsynaptic currents (sPSCs), indicating enhanced neuronal excitation (Figure 6B). In contrast to untreated neurons, application of baclofen did not reduce the frequency of sPSCs in OGD-treated neurons, suggesting insufficient GABA_B_ receptor-mediated inhibition after ischemic stress. However, the application of R2-Pep to OGD-stressed neurons significantly reduced the frequency of sPSCs (Figure 6B’) and significantly increased baclofen-induced inhibition of current amplitudes (Figure 6B’’). These findings indicate that restoration of GABA_B_ receptor-mediated inhibition in neurons subjected to OGD reduces neuronal overexcitation.

### 3.7 R2-Pep Reduces Oxygen Glucose Deprivation-Induced Neuronal Death

**FIGURE 7:**
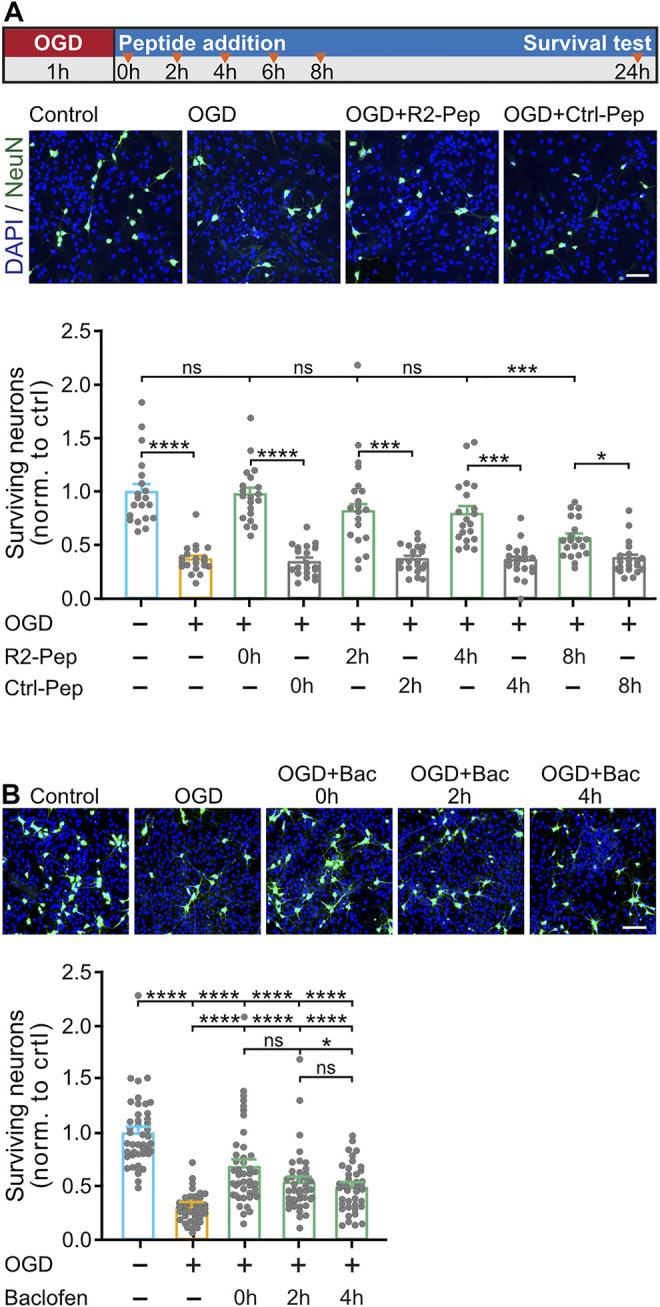
R2-Pep limits OGD-induced neuronal death and is more effective than baclofen. A, OGD-stressed cultures were treated with R2-Pep or Crtl-Pep immediately (0 h), 2, 4, 6 or 8 h after the stress. After 24 h the cultures were immunostained for neurons (NeuN, green) and total cells (DAPI, blue). Top: scheme of experimental design. Middle: representative images (scale bar: 100 μm). Bottom: Quantification of surviving neurons. The value of the untreated cultures served as control. The data represent the mean ± S.E.M. of 20 frames (fields of view) for each experimental condition derived from 2 independent experiments. ns: *p* > 0.05; *, *p* < 0.05; ***, *p* < 0.001; ****, *p* < 0.0001; Brown-Forsythe/Welch one-way ANOVA followed by Dunnett’s T3 multiple comparison test. B, OGD-stressed cultures were treated with baclofen immediately (0 h), 2 h or 4 h after the stress. After 24 h the cultures were immunostained for neurons (NeuN, green) and total cells (DAPI, blue). Top: representative images (scale bar: 100 μm). Bottom: Quantification of surviving neurons. The value of the untreated cultures served as control. The data represent the mean ± S.E.M. of 40–45 frames for each experimental condition derived from 3 independent experiments. ****, *p* < 0.0001; one-way ANOVA with Tukey’s multiple comparisons test.

Finally, we tested whether preventing downregulation of GABA_B_ receptors by R2-Pep is neuroprotective. Co-cultures of neurons and glia cells were submitted for 1 h to OGD and treated thereafter with R2-Pep or Ctrl-Pep at different time intervals (see Figure 7A for the experimental outline). To monitor loss of neurons, cultures were stained for the neuronal marker protein NeuN and with DAPI for total cells (the vast majority of cells in the cultures are glia cells, which require much longer exposure to OGD to induce death (Benavides et al., 2005)).

In OGD-stressed cultures, a substantial loss of neurons (37 ± 3% of control) was observed after 24 h, indicating considerable neuronal death (Figure 7A). Loss of neurons was completely blocked upon administration of R2-Pep immediately after the stress (98 ± 6% of control) and strongly attenuated (not statistically significant different from control) upon treatment after 2 h (82 ± 7% of control) and 4 h (80 ± 7% of control). The neuroprotective activity dropped to 57 ± 4% of control when cultures were treated with R2-Pep 8 h after the OGD-stress.

Next, we tested whether the neuroprotective activity of R2-Pep is superior to that of the GABA_B_ receptor agonist baclofen. Baclofen exhibits some level of neuroprotection under ischemic conditions (Lal et al., 1995; Babcock et al., 2002; Zhang et al., 2007; Han et al., 2008; Xu et al., 2008; Zhou et al., 2008; Liu et al., 2015; Fu et al., 2016; Lu et al., 2016; Hleihil et al., 2021), but this might be suboptimal in view of the downregulation of the receptors. As expected, compared to R2-Pep, baclofen showed considerably less neuroprotection at all time points tested (0 h: 68 ± 6%, 2 h: 54 ± 5% and 4 h: 50 ± 3% of control) (Figure 7B).

## 4 Discussion

Stressful conditions, such as cerebral ischemia, impair ER function which causes the accumulation of proteins in the ER. This triggers the activation of ER stress response pathways to restore proper function of the ER (Hetz 2012; Kaneko et al., 2017). When the functions of the ER cannot be restored and ER response pathways can no longer limit the damage, apoptosis is induced. One important ER stress-induced apoptotic pathway is triggered by upregulation of the transcription factor CHOP, whose expression level is otherwise marginal under normal physiological conditions (Oyadomari and Mori 2004; Yang et al., 2017; Hu et al., 2018). Apart from functioning as an apoptosis-inducing transcription factor, CHOP has also been shown to interact with GABA_B_ receptors (Sauter et al., 2005). This interaction obstructs the assembly of the GABA_B_ receptor subunits GABA_B1_ and GABA_B2_ in the ER, prevents the ER exit of GABA_B1_/GABA_B2_ receptor complexes, thereby blocking the supply of new receptors to the cell surface (Maier et al., 2014). This mechanism downregulates GABA_B_ receptors from the cell surface since constitutive internalization and degradation of the receptors continues but the supply of new receptors is blocked. The resulting loss of GABA_B_ receptor-mediated neuronal inhibition is another detrimental effect of CHOP after ischemic stress which fosters neuronal overexcitation and excitotoxic neuronal death.

In this study, we reasoned that blocking the GABA receptor/CHOP interaction after ischemic stress would restore normal GABA_B_ receptor expression and function, which might limit excitotoxic neuronal death. To test this hypothesis, we designed a cell-permeable synthetic peptide (R2-Pep) comprising a short amino acid sequence located within the C-terminal domain of GABA_B2_, which we formerly identified as part of the CHOP interaction site (Sauter et al., 2005). To mimic ischemic conditions *in vitro*, we used oxygen and glucose deprivation (OGD) of primary neurons co-cultured with glia cells. Co-cultures of neurons and glia cells were chosen because they closely mimic the brain situation, as glia cells support neurons and make them more resistant to stressful condition like OGD (Gouix et al., 2014; Liu and Chopp 2016). Glia cells have been reported to express GABA_B_ receptors. For instance, in astrocytes, activation of GABA_B_ receptors induce Ca^2+^ transients resulting in astrocytic glutamate release (Meier et al., 2008). Thus, we cannot rule out a contribution of glial GABA_B_ receptors to the effects of R2-Pep treatment. However, in our neuron/glia cultures, expression of GABA_B_ receptors on glia cells appears to be rare or at a very low levels not detectable with the GABA_B_ receptor antibodies used in this study. In addition, the conditions used (OGD for 1 h) hardly upregulates CHOP in glia cells, which are much more resistant to ischemic stress as compared to neurons. At least 4 h of OGD is required to induce upregulation of CHOP in astrocytes (Benavides et al., 2005). Therefore, it is rather unlikely that R2-Pep affects GABA_B_ receptor expression in glia cells.

We found that exposure of neuron/glia cultures to 1-h OGD upregulated CHOP expression, reduced GABA_B1_/GABA_B2_ heteromers and downregulated GABA_B_ receptors from the cell surface. As hypothesized, the interfering peptide R2-Pep restored normal heterodimerization and cell surface expression of the receptors. This was reflected by recovered GABA_B_ receptor function (i.e., baclofen-induced potassium currents) and a reduction of OGD-induced increased neuronal activity (i.e., reduction of enhanced spontaneous postsynaptic currents). The complete restoration of GABA_B_ receptor expression to normal levels was unexpected since the interaction of CHOP with GABA_B_ receptors is only one pathway contributing to the downregulation of GABA_B_ receptors under ischemic/excitotoxic conditions. An additional main pathway is the aberrant sorting of internalized receptors to lysosomal degradation (Guetg et al., 2010; Maier et al., 2010; Terunuma et al., 2010; Kantamneni et al., 2014). However, our results suggest that interfering with the CHOP/GABA_B_ receptor interaction is sufficient to normalize cell surface expression of the receptors by enabling the supply of new receptors from the ER.

Furthermore, treatment with R2-Pep also normalized OGD stress-induced CHOP expression back to control levels. The expression of CHOP is tightly controlled by proteasomal degradation. Monomeric CHOP is constitutively polyubiquitinated and degraded by the proteasome, whereas dimerization via their leucine zippers stabilizes CHOP (Hattori et al., 2003). CHOP bound to R2-Pep is most likely recognized as monomer and rapidly degraded. This view is supported by our finding that blocking proteasomal degradation prevents the R2-Pep induced downregulation of CHOP. Another mechanism downregulating stress-induced CHOP expression by R2-Pep might be directly related to the normalized GABA_B_ receptor expression and function. Activation of GABA_B_ receptors by baclofen has been shown to reduce enhanced CHOP expression induced by hypoxia in retinal ganglion cells (Fu et al., 2016). It is currently unclear whether directly interfering with the GABA_B_/CHOP interaction or indirectly via the downregulation of CHOP is the primary cause for the R2-Pep-induced normalization of GABA_B_ receptor expression and function. However, the ability of R2-Pep to reduce CHOP expression to normal physiological levels offers the opportunity to prevent CHOP-mediated cell death in pathological conditions primary unrelated to GABA_B_ receptors such as diabetes (Oyadomari et al., 2002), asthma (Wang et al., 2017) or Alzheimer’s (Prasanthi et al., 2011).

GABA_B_ receptors are organized in multiprotein signalling complexes comprising the receptor, G-proteins, KCTD proteins, effector proteins and other associated proteins (Schwenk et al., 2010; Schwenk et al., 2016). We, therefore, assumed that the inwardly rectifying K^+^ channel K_ir_3.2, which is one of the main postsynaptic effectors, is co-regulated with the GABA_B_ receptors under ischemic conditions. Indeed, we found that K_ir_3.2 was downregulated to a similar extent as GABA_B_ receptors after OGD stress and application of R2-Pep normalized their expression. This result agrees with previous findings showing the association and co-regulation of GABA_B_ receptors with inwardly rectifying K^+^ channels under different physiological or pathological conditions (David et al., 2006; Fernandez-Alacid et al., 2009; Padgett et al., 2012; Hearing et al., 2013).

Since GABA_B_ receptors are involved in the regulation of virtually all main brain functions and can regulate gene expression, it would be very interesting to analyze the effect of R2-Pep after an ischemic insult on gene expression on a genome wide level. In addition to identifying negative off-target effects of R2-Pep, this would also generate valuable information of the pathways affected by the restoration of GABA_B_ receptors.

In line with a neuroprotective function of GABA_B_ receptors in *in vivo* models of cerebral ischemia (Lal et al., 1995; Babcock et al., 2002; Zhang et al., 2007; Han et al., 2008; Xu et al., 2008; Zhou et al., 2008; Liu et al., 2015; Fu et al., 2016; Lu et al., 2016; Hleihil et al., 2021), we found that treatment of cultures with R2-Pep for up to 4 h after OGD stress largely prevented neuronal death. We also found that R2-Pep has a considerably higher neuroprotective activity than the GABA_B_ receptor agonist baclofen. This was expected because of the downregulation of the receptors after OGD. Based on the observation that activation of GABA_B_ receptors is neuroprotective in *in vivo* models and the finding that CHOP deficient mice show a reduced level of neuronal death after an ischemic insult (Tajiri et al., 2004), it is very likely that the neuroprotective activity of R2-Pep we observed in our *in vitro* model also translates to *in vivo* models. The main challenge for peptide therapeutics targeting the brain is to cross the blood-brain barrier and escape degradation by peptidases and rapid elimination from the circulation. However, there are currently promising nanoparticle-based drug delivery approaches available, that are reported to overcome these problems (Han et al., 2016; Li et al., 2019; Pinheiro et al., 2020). This issue needs to be addressed in future experiments.

Since GABA_B_ receptors are ubiquitously expressed in the brain and are involved in virtually all main brain functions, global activation of GABA_B_ receptors by its orthosteric agonist baclofen is associated with numerous side effects when given systemically (Slonimski et al., 2004; Brennan and Whittle 2008). For this reason, baclofen is largely restricted to the treatment of severe spasticity via intrathecal application (Slonimski et al., 2004). In contrast to global activation of GABA_B_ receptors, targeting a specific disease-relevant aberrant protein-protein interaction, such as the GABA_B_ receptor/CHOP interaction, offers the opportunity to develop highly specific therapeutics that preferentially target the diseased cells and might leave healthy cells largely unaffected. This is expected to result in safe treatments with favourable side effect profiles.

## Data Availability

The original contributions presented in the study are included in the article/supplementary material, further inquiries can be directed to the corresponding author.
